# Preparation and Characterization of Biodegradable κ-Carrageenan Based Anti-Bacterial Film Functionalized with Wells-Dawson Polyoxometalate

**DOI:** 10.3390/foods11040586

**Published:** 2022-02-18

**Authors:** Feng Zhou, Dehua Wang, Jiawen Zhang, Jing Li, Danning Lai, Shaoling Lin, Jiamiao Hu

**Affiliations:** 1 Engineering Research Centre of Fujian-Taiwan Special Marine Food Processing and Nutrition, Ministry of Education, Fuzhou 350002, China; fengzhouzftw@163.com (F.Z.); wangdehua105@163.com (D.W.); 3210910058@fafu.edu.cn (J.Z.); lijing0825@fafu.edu.cn (J.L.); 3200910025@fafu.edu.cn (D.L.); shaoling.lin@fafu.edu.cn (S.L.); 2 College of Food Science, Fujian Agriculture and Forestry University, Fuzhou 350002, China; 3 Key Laboratory of Marine Biotechnology of Fujian Province, Institute of Oceanology, Fujian Agriculture and Forestry University, Fuzhou 350002, China

**Keywords:** polyoxometalate, kappa-carrageenan, anti-bacterial film, biodegradable

## Abstract

In the present study, an anti-bacterial film (Carr/POM film) was prepared through the incorporation of Wells-Dawson polyoxometalate K_6_[Mo_18_O_62_P_2_] into κ-carrageenan-based polymers using the tape-casting method. The mechanical properties, thermal stability, and morphology of the prepared film were characterized. The obtained results showed that incorporation of K_6_[Mo_18_O_62_P_2_] significantly affected the morphology and structure of the films. Moreover, the polyoxometalate-based film demonstrated desirable bactericidal activity against *Escherichia coli* (Gram-negative) and *Staphylococcus aureus* (Gram-positive). Carr/POM (@8 mg/mL) film resulted in an obvious inhibition zone around the film in Kirby-Bauer disk diffusion test, which could also remove 99% of *S. aureus* and *E. coli* on plastic, glass, and stainless steel. In addition, this anti-bacterial film also demonstrated good biodegradability, which could be decomposed in soil in around 1 week. In conclusion, the polyoxometalate-based film showed good anti-bacterial property against food-borne pathogenic microbes, suggesting the prepared film has great potential to be developed as promising food packaging.

## 1. Introduction

Microbial contamination poses a substantial threat to human health and causes huge economic losses in food industry [1,2]. According to a report from the World Health Organization (WHO), more than 600 million people worldwide suffer from microbial foodborne illnesses annually, resulting in a more than $95 billion loss [3]. Packaging is an essential component in the food industry, which can be used to maintain the integrity, quality, freshness, and safety of foods. It could protect the food products from microbial contamination, appearance damage, changes in color and texture, and reduce the incidence of foodborne diseases [4].

In the past decades, development of novel anti-bacterial food packaging has drawn great attention of scholars [5]. Especially, with the rise of ecological concerns, biodegradable food packaging made from various biopolymers appeals to many researchers [6,7]. Among the commonly used biopolymers, carrageenan is abundant in nature. Particularly, κ-carrageenan has been widely adopted for the preparation of bio-based packaging materials because of its excellent film-forming properties. Meanwhile, a number of studies have shown incorporating anti-bacterial agents into carrageenan-based films is a promising strategy to improve the anti-bacterial effect [8]. For instance, Nouri et al. [9] reported the κ-carrageenan films containing 3% zataria multiflora plant extract could effectively inhibit the growth and reproduction of *Escherichia coli* (*E. coli*), *Staphylococcus aureus* (*S. aureus*), *Bacillus cereus* (*B. cereus*), and *Pseudomonas aeruginosa* (*P. aeruginosa*). Moreover, Duan et al. [10] also reported that the κ-carrageenan/konjac glucomannan film containing 7% TiO_2_ nano-particles showed an obvious anti-bacterial effect on *Penicillium viridicatum*, and the anti-bacterial rate reached 79%. Furthermore, anti-bacterial packaging is one of the most important active packaging, which can be used to preserve the quality and prolong the shelf life of food products. For example, Abdillah et al. [11] successfully extended the shelf life of cherry tomatoes to 10 days by iota-carrageenan-based film containing arrowhead starch. Zhang et al. [12] also reported that polylactic acid film containing chitosan could delay spoilage of fish fillets for 3 days by inhibiting the growth and reproduction of *E. coli* and *S. aureus*.

Polyoxometalates (POMs) are a large group of discrete anionic metal-oxygen clusters formed by the coordination of early transition metal atoms (usually in their highest oxidation states) with oxygen atoms [13]. According to the ratio of center atoms to the hetero atoms, the most prominent and commonly used polyoxometalates can be divided into following types: Wells-Dawson, Keggin, Anderson, Waugh, Silverton, Lindqvist, Weakley, Strandberg, Finke, and Preyssle [14]. The decades of polyoxometalates research have revealed their great antimicrobial potential [15,16,17,18]. Particularly, Wells-Dawson and Keggin polyoxometalates were found to be most potent in terms of sensitizing methicillin-resistant *S. aureus* strains SR3605 and ATCC43300 among 76 tested POMs [19]. Moreover, in another trial, Inoue et al. [20] reported Wells-Dawson polyoxometalates has stronger effects than Keggin polyoxometalates in terms of sensitizing methicillin-resistant *S. aureus*. It is also worth mentioning that besides its bactericidal activity, Wells-Dawson polyoxometalates also showed great potential for food (especially the fruits and vegetables) preservation due to its inhibitory effects on enzymatic browning. For instance, Lampl et al. demonstrated that Wells-Dawson polyoxometalates could serve as tyrosinase inhibitors and significantly slow down the browning of mushroom [21]. Similarly, Hu et al. also synthesized a Wells-Dawson phosphotungstic polyoxometalate and confirmed its inhibitory effects on mushroom tyrosinase [22]. Recent research also showed the polyoxometalates could be used to prevent browning of apple slices [23] and lotus root slices [24]. Therefore, Wells-Dawson polyoxometalates might be very promising anti-bacterial agents for food preservation.

However, there is a notable paucity of literature describing the anti-bacterial films based on the Wells-Dawson polyoxometalates. Therefore, in this study, research was carried out to prepare an anti-bacterial film by incorporating Wells-Dawson polyoxometalate K_6_[Mo_18_O_62_P_2_] into κ-carrageenan-based polymers with characterizing its physical and mechanical properties. In addition, the anti-bacterial and degradation properties of the film were also evaluated.

## 2. Materials and Methods

### 2.1. Materials and Methods

κ-carrageenan and glycerol were obtained from Sinopharm Chemical Reagent Co., Ltd. (Shanghai, China) Polyoxometalate (K_6_[Mo_18_O_62_P]) were synthesized according to the previous literature [25]. *Escherichia coli* (*E. coli*) and *Staphylococcus aureusin* (*S. aureus*) were purchased from China of Industrial Culture Collection (Beijing, China). LB broth and LB broth with agar were ordered from Solarbio.

### 2.2. Preparation of Carr/POM Film

κ-carrageenan powder (1%, *w*/*v*) and glycerol (0.05%, *v*/*v*) were dissolved in sterile water at 80 °C and stirred magnetically for 30 min to obtain a clear, transparent, and uniform film-forming solution. Wells-Dawson polyoxometalate K_6_[Mo_18_O_62_P_2_] powder was dissolved in sterile water and then mixed with the film-forming solution with magnetic stirring at 60 °C for 20 min to obtain Carr/POM film-forming solutions at different concentrations (0–8 mg/mL). Finally, the films were prepared by the tape-casting method and dried in an oven with air circulation at 45 °C for 24 h. The prepared films were stored at room temperature (25 ± 1 °C) and relative humidity of 50%.

### 2.3. Measurement of the Film Thickness

The film thickness was measured using a spiral micrometer (Guanglu 211-101). Randomly selected 5 points on each film were determined and the obtained values were averaged as the film thickness.

### 2.4. Measurement of Water Content in the Film

The film water content (WC) was measured by a TC-SFY-30 infrared fast moisture tester (Beijing Tongde venture Technology Co., Ltd., Beijing, China). The film was placed into the Infrared fast moisture tester and dried to constant weight. The weight loss was recorded by the instrument as the water content of the film. Each film was measured three times before the results were averaged.

### 2.5. Measurement of the Tensile Strength of the Film

The tensile strength (the maximum force value taken at rupture) of film was measured according to the standard method described in ASTM-D638 [26]. The film samples were cut into 1 cm × 5 cm in size and determined by fixing film strip on grips of AGS-J texture analyzer (Shimadzu, Kyoto, Japan) with a crosshead speed of 0.5 mm/s and distance of 30 mm. Experiments were conducted in triplicate, and the tensile strength was calculated as force (N) divided by the cross-sectional area (m^2^).

### 2.6. Measurement of Color and Transparency of the Film

The transparency of film was measured as previously reported by Sood et al. [27]. The film samples with a size of 1 × 5 cm were attached to the inner wall of cuvette. The absorbance value (at 600 nm) was measured using a UV-Vis spectrophotometer (Mapada Instrument Co., Ltd., Shanghai, China). Experiments were conducted in triplicate. The opacity of the film is calculated as absorbance at 600 nm divided by the film thickness (mm)

The surface color indices (L, a, b values) of the film were measured using a colorimeter (CM-5, Konica Minolta), which was calibrated using a white color standard. The total color difference (∆E) was calculated using the ∆L*, ∆a*, and ∆b* values as follows:

(1) $$\Delta E=\sqrt{{\left(\Delta L\right)}^{2}+{\left(\Delta a\right)}^{2}+{\left(\Delta b\right)}^{2}}$$

### 2.7. Scanning Electron Microscopy (SEM)

SEM was used to determine the surface microstructure of the films. In brief, the film was cut into a round piece with a diameter of 6 mm. The pieces mounted on a cylindrical aluminum specimen holder were scrutinized under a JEOL JSM-6380lv scanning electron microscope (JEOL Ltd., Tokyo, Japan).

### 2.8. Fourier-Transform Infrared Spectroscopy (FT-IR)

The molecular interaction between K_6_[Mo_18_O_62_P_2_] and κ-carrageenan was examined by FT-IR. In short, the dried film was mixed and ground with dried potassium bromide powder, and the tablet was prepared by the tablet-pressing method. The FT-IR spectra in the range of 500–4000 cm^−1^ were then recorded and analyzed.

### 2.9. Thermogravimetric Analysis

The thermal stability of Carr/POM film was tested by DSC/DTA-TG (STA449C/6/G, NETZSCH Group). In brief, 3 mg of Carr/POM film was heated from 25 °C up to 600 °C at a heating rate of 10 °C/min under a nitrogen gas flow.

### 2.10. Determination of Bactericidal Activity of Carr/POM Film

#### 2.10.1. Kirby-Bauer Disc Diffusion Method

The experiment was carried out as reported by Yu et al. [28]. In short, the bacteriostatic film was cut into small discs with a diameter of 6 mm. Then, they were pasted on the surface coated with *E. coli* and *S. aureus* culture medium respectively. They were cultured for for 24 h at 37 °C, removed, their bacteriostatic diameter was recorded, and photos were taken.

#### 2.10.2. Carrier Surface Disinfection Test

The experiment was performed using the method described by MSc et al. [29]. *E. coli* and *S. aureus* bacterial solution were inoculated dropwise on stainless steel, glass, and plastic respectively, and then Carr/POM anti-bacterial film was applied onto the carrier surface for 30 min at 37 °C. The bacteria on both carrier surface and film were recovered and enumerated using the plate colony count method.

### 2.11. Determination of Film Degradability in Soil

The film degradability was measured by adapting the experimental method reported by Rech et al. [30]. In brief, the prepared films were wrapped with gauze and buried in the soil with a depth of 10 cm at 25 °C, 80% humidity, and were taken out and photographed every day to observe the degree of degradation.

### 2.12. Statistical Analysis

All experimental measurements were conducted at least in triplicate. The quantitative results were presented as mean ± standard deviation. SPSS 26 software (IBM, Chicago, IL, USA) was used to analyze the significance by Duncan multiple-range test (* *p* < 0.05).

## 3. Results and Discussion

### 3.1. The Effects of Polyoxometalate Concentration on the Thickness of the Film

**Figure 1:**
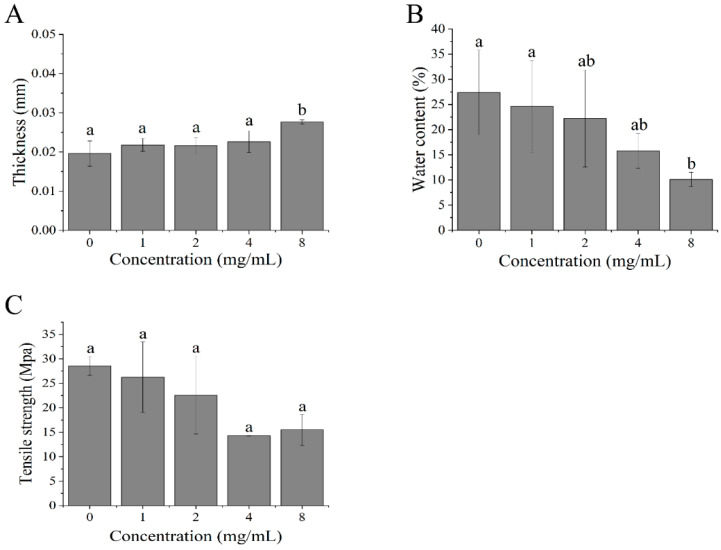
Effect of polyoxometalate (POM) concentration on thickness (**A**), water content (**B**), and tensile strength (**C**) of Carr/POM anti-bacterial films. Different lowercase letters represent significant differences, and the same lowercase letters represent no significant differences (*p* < 0.05).

The effects of polyoxometalate incorporation on the thickness of the Carr/POM film were investigated. As illustrated in Figure 1A, the thickness of κ-carrageenan film without polyoxometalate incorporation was 0.020 ± 0.003 mm, while the addition of polyoxometalate at low concentration (≤4 mg/mL) had little impact on the thickness of the films. However, further addition of polyoxometalate (8 mg/mL) resulted in a significant increase in the film thickness (* *p* < 0.05). This may be attributed to increases in solid content or the internal gap between the polymer chain and glycerol in the film matrix as a consequence of polyoxometalate incorporation [31]. Indeed, this finding is consistent with a number of previous studies [9,32]. For example, the incorporation of bee pollen extract [33] and hazelnut extract [34] into κ-carrageenan-based films also resulted in the increase in film thickness.

### 3.2. The Effects of Polyoxometalate Concentration on the Water Content of the Film

As illustrated in Figure 1B, compared with the control group, the addition of polyoxometalate in the prepared film led to an overall declining trend of the water content. The κ-carrageenan film showed the highest water content (27.4% ± 8.4%), while the lowest water content (10.1% ± 1.4%) was recorded in Carr/POM (@8 mg/mL) film among all tested films. The possible causes of decreases in water content of Carr/POM films might be newly-formed hydrogen bonds between polyoxometalate and κ-carrageenan/glycerol, which reduced the formation of hydrogen bonds between κ-carrageenan/glycerol and water, and therefore decreased the water content of the film [35,36].

### 3.3. The Effects of Polyoxometalate Concentration on the Tensile Strength of the Film

Tensile strength is an important mechanical property of packing materials [37]. Figure 1C demonstrated that the tensile strength of prepared films showed a decreasing trend with increasing polyoxometalate incorporation. The decreased mechanical properties of the Carr/POM films may result from the formation of micropores on the film matrix as well as the decrease in the intermolecular and intramolecular interactions between κ-carrageenan polymer chains upon incorporation of polyoxometalates [32].

### 3.4. The Effects of Polyoxometalate Concentration on the Color and Opacity of the Film

**Figure 2:**
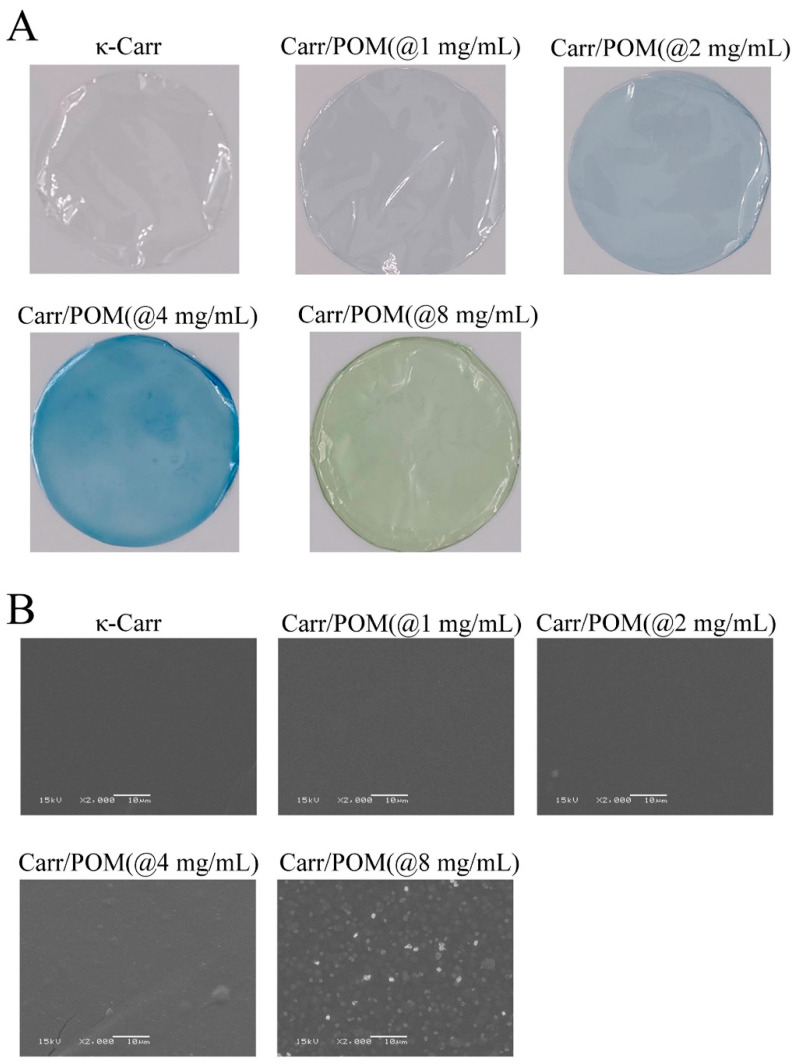
Morphology (**A**) and SEM micrographs (**B**) of neat carrageenan film and Carr/POM anti-bacterial films incorporated with K_6_[Mo_18_O_62_P_2_] at different concentrations.

Surface color and opacity of Carr/POM films are presented in Table 1 and Figure 2. The film without polyoxometalate incorporation was nearly transparent without color, while the Carr/POM films showed blue and light green (Figure 2A), as reflected by the increases in the L and b values of Carr/POM films (Table 1). Notably, the film with 8 mg/mL polyoxometalate incorporation showed less blue than films with polyoxometalate at lower concentrations. This phenomenon might result from the different pH values of the forming solution containing K_6_[Mo_18_O_62_P_2_] at different concentrations since research has already revealed that polyoxometalates could be pH-responsive dyes [38].

Meanwhile, the difference in transparency between neat carrageenan film and polyoxometalate incorporated films was also obvious. In general, the addition of polyoxometalate significantly increased the opacity of the film (* *p* < 0.05). This may be because the addition of the polyoxometalates hinders the transmission of light [39].

### 3.5. The Effects of Polyoxometalate Concentration on the Microstructures of the Film

The apparent morphology of the film was next determined by scanning electron microscope. As illustrated in Figure 2B, the surface of film without polyoxometalate was smooth and uniform, indicating κ-carrageenan has good compatibility with glycerol without phase separation. Similarly, SEM images also showed no obvious difference between the film surface of Carr/POM (@1 & 2 mg/mL) films and that of neat carrageenan film, suggesting that the polyoxometalate could bind to the film matrix well at low concentrations (≤2 mg/mL). However, with the further increase in polyoxometalate incorporation, increased surface roughness or even some cracks appeared on the surface of the film, especially at high concentrations (8 mg/mL). This phenomenon may result from the aggregation of K_6_[Mo_18_O_62_P], making the surface less smooth. Similar results were also reported, i.e., that incorporation of anti-bacterial agents (e.g., lycium barbarum extract [40], mulberry anthocyanin extract [26]) into the κ-carrageenan films obviously affect the surface smoothness. Indeed, these observations might also support the findings that incorporation of polyoxometalates led to the increase in film thickness and decrease in tensile strength.

### 3.6. FTIR Spectroscopic Analysis of Carr/POM Film

**Figure 3:**
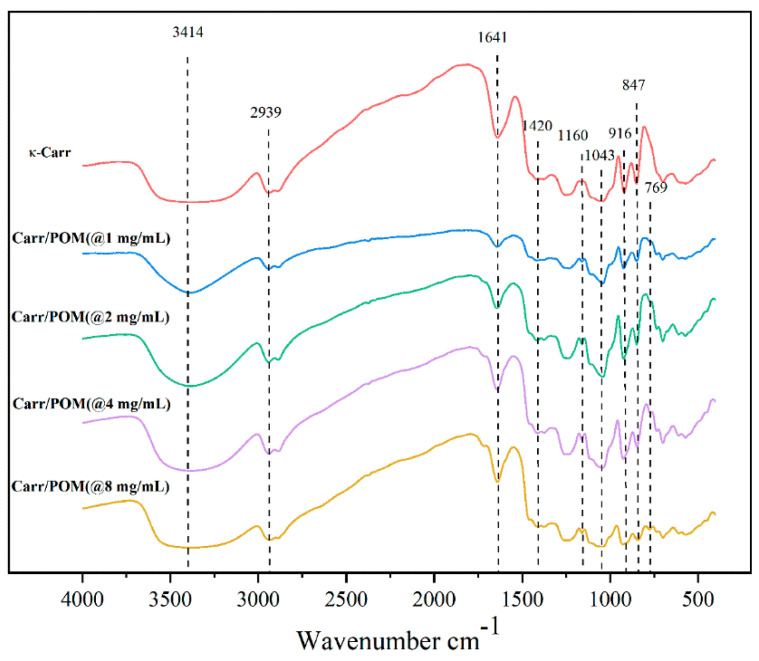
Fourier-transform infrared spectroscopy (FT-IR) of neat carrageenan film and Carr/POM anti-bacterial films incorporated with K_6_[Mo_18_O_62_P_2_] at different concentrations.

FTIR spectroscopic analysis was performed to analyze the intermolecular interactions of Carr/POM films, as illustrated in Figure 3. The FTIR spectrum of carrageenan film has multiple characteristic peaks in the range of 500–4000 cm^−1^. At 3414 cm^−1^, a wide and large absorption peak was attributed to the stretching vibration of O-H-O [26]. The absorption peaks at 3114 cm^−1^, 2939 cm^−1^, 1160 cm^−1^, 1043 cm^−1^, 916 cm^−1^, and 847 cm^−1^ were due to O-H stretching, C-O stretching vibration, sulfate bonds, glycosidic bonds in C-O mode, C-O stretching vibration, and C-O-SO_3_, respectively [40,41]. Additionally, the peaks at 1641 cm^−1^ and 1420 cm^−1^ represented the bending of bound water. When introducing the polyoxometalate into the film, changes in position and intensity of the FT-IR peak were observed. For instance, the new appeared peak at 769 cm^−1^ was due to Mo-O_c_-Mo stretching vibration. In addition, it could also be found that the absorption peaks of Carr/POM(@8 mg/mL) films changed from narrow to wide at 1043 cm^−1^ and 916 cm^−1^, which might be caused by Mo-O_d_ and Mo-O_b_-Mo, respectively [42].

### 3.7. Thermogravimetric Analysis of Carr/POM Anti-Bacterial Film

**Figure 4:**
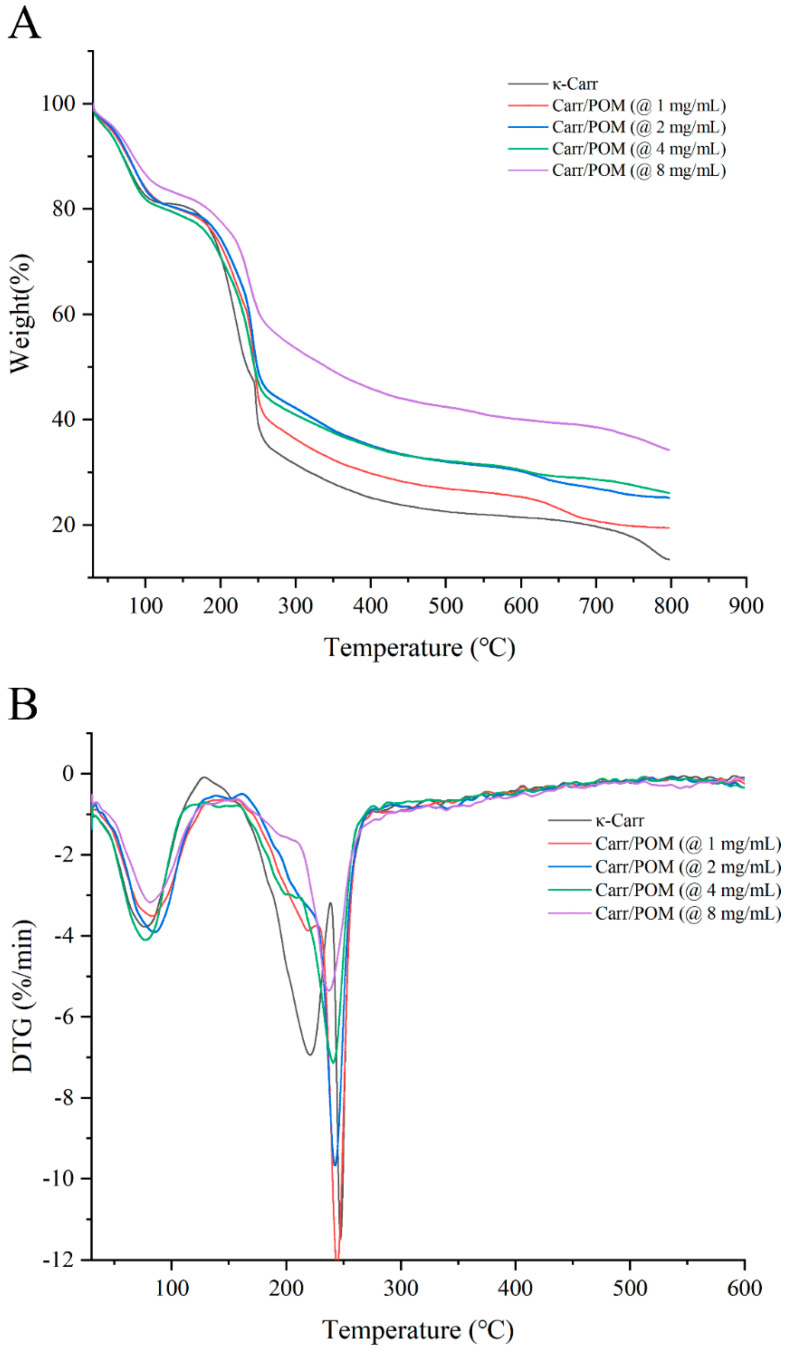
TGA (**A**) and DTG (**B**) curves of neat carrageenan film and Carr/POM anti-bacterial films incorporated with K_6_[Mo_18_O_62_P_2_] at different concentrations.

The effect of polyoxometalate concentration on the thermal stability of the films was analyzed by thermogravimetric analysis. The TGA and DTG profiles of κ-carrageenan control film and Carr/POM films are shown in Figure 4. All films exhibited three thermal decomposition stages. The first thermal decomposition stage ranging from 30 to 90 °C is due to the hydrophilicity of carrageenan, resulting in the evaporation of the water [43]. The second thermal decomposition stage was at 130–220 °C, mainly caused by the degradation of glycerol [44]. Notably, the weight loss of Carr/POM (at 2 and 4 and 8 mg/mL) films in this stage became not obvious. The third thermal decomposition stage occurred at 210–250 °C, which mainly results from the thermal degradation of carrageenan matrix [10]. In general, the TGA curves of κ-carrageenan control film and Carr/POM films showed a high similarity, though the second and third stages of thermal decomposition of Carr/POM films occurred at a relatively lower temperature compared with the control film, revealing that polyoxometalates might reduce thermal stability of the films resulting from the decreased interactions between polyoxometalates/glycerol and the κ-carrageenan matrix [45]. Indeed, these observations might also support the finding that incorporation of polyoxometalates led to the decrease in tensile strength of the films.

### 3.8. Bactericidal Activity of Carr/POM Anti-Bacterial Film

**Figure 5:**
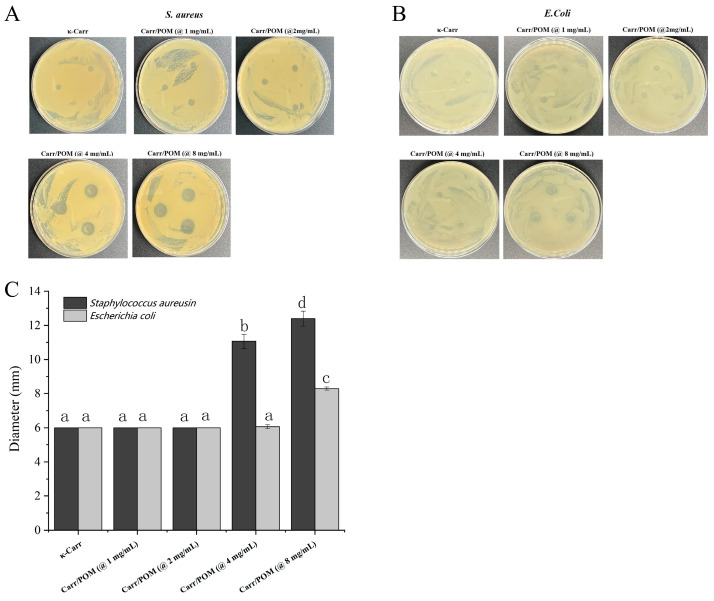
The bacteriostatic effects of the Carr/POM anti-bacterial films. The representative images of bacteriostatic circle of Carr/POM anti-bacterial films against *S. aureus* (**A**) and *E. coli* (**B**); the average diameters of bacteriostatic circle of the Carr/POM anti-bacterial films incorporated with K_6_[Mo_18_O_62_P_2_] at different concentrations (**C**). Different lowercase letters represent significant differences, and the same lowercase letters represent no significant differences (*p* < 0.05).

As shown in Figure 5, the κ-carrageenan film as well as Carr/POM (at 1 and 2 mg/mL) films showed little bactericidal effects against *E. coli* and *S. aureus.* In contrast, the Carr/POM (@4 mg/mL) resulted in an obvious inhibition zone around the film in the Kirby-Bauer disk diffusion test. The inhibition zone diameter further enlarged when the polyoxometalate concentration increased to 8 mg/mL. Meanwhile, the results obtained from the Kirby-Bauer disk diffusion test also suggested that the killing effects of Carr/POM films against *S. aureus* were obviously stronger than *E. coli*. Indeed, the outer membrane of Gram-negative bacteria has been proven to be the main reason for resistance to a wide range of antibiotics (e.g., macrolides, glycopeptides, rifamycins, etc.) [46,47,48], which might also explain the difference in the killing effects of Carr/POM films against *E. coli* and *S. aureus*. Notably, it was also observed that the films containing polyoxometalates faded upon incubation with bacteria for 24 h, therefore, we speculated that the release of embedded polyoxometalates may greatly contribute to the antibacterial activity of the films.

**Figure 6:**
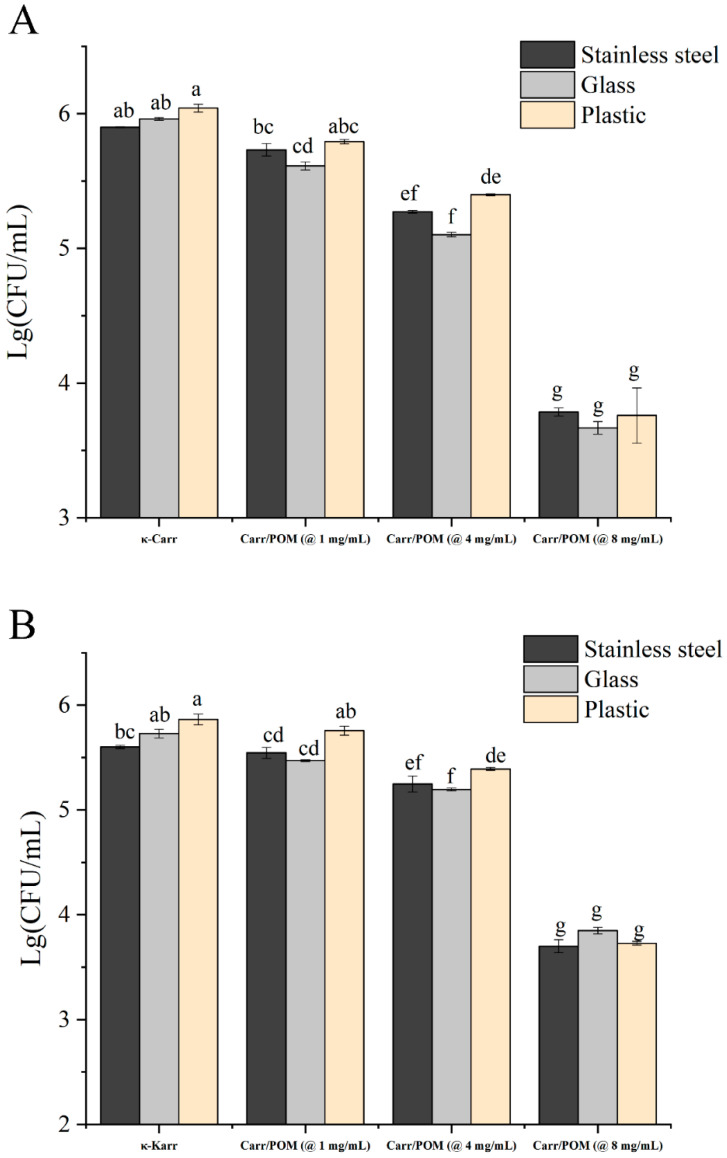
Bactericidal activity of Carr/POM films against *S. aureus* (**A**) and *E. coli* (**B**) on three different surfaces (stainless steel, glass, and plastic). Different lowercase letters represent significant differences, and the same lowercase letters represent no significant differences (*p* < 0.05).

Additionally, the application potential of Carr/POM films to clean the bacterial contaminated surface was explored. As shown in Figure 6, the Carr/POM anti-bacterial films showed obvious bactericidal effects on *S. aureus* and *E. coli* on both stainless steel, glass, and plastic. Compared with κ-carrageenan film, the sterilization rate of Carr/POM (at 8 mg/mL) film against *E. coli* and *S. aureus* on stainless steel, glass, and plastic could reach 99%. Furthermore, similar to the observation in Kirby-Bauer disk diffusion test, the Carr/POM exerted stronger killing effects against *S. aureus* compared with *E. coli*; while the types of carriers also influenced the anti-bacterial activity of the films (glass > plastic > stainless steel). This may be due to difference in the adhesion of microorganisms on various material surfaces [49]. For instance, smooth surfaces (e.g., glass) often showed low level of bacterial adhesion, while rough surfaces may enhance the bacterial adhesion and reduce the contact with the anti-bacterial film [50,51].

### 3.9. Degradability of Carr/POM Anti-Bacterial Film in Soil

**Figure 7:**
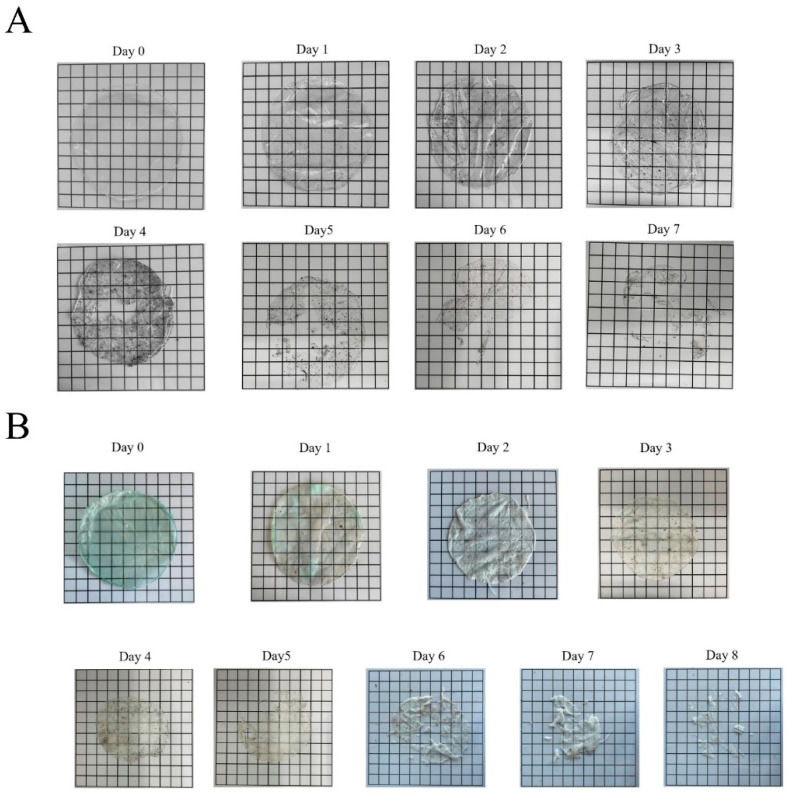
Degradation of neat carrageenan film (**A**) and Carr/POM film (**B**) in soil.

The biodegradability of the Carr/POM film was next explored. As shown in Figure 7A, the κ-carrageenan film maintained intact in the first two days, gradually degraded after day 3, and finally become fully degraded on the day 7. Meanwhile, the Carr/POM (at 8 mg/mL) film showed no breakage but gradually faded in the first two days; afterwards, obvious cracks appeared, and the film finally completely degraded on the day 8 (Figure 7B). These results suggested the Carr/POM films possess good biodegradability in soil, highlighting their potential application as an environmentally friendly anti-bacterial material. This observation was consistent with previous findings that the carrageenan-based films possess good biodegradability [52,53]. For instance, an edible film fabricated using iota-carrageenan and arrowroot starch could be completely degraded in a week [11], while and κ-carrageenan films containing curcumin-β-cyclodextrin could be degraded on day 7 [8].

## 4. Conclusions

For a long time, polyoxometalates have been favored by researchers because of their remarkable anti-bacterial properties, which are promising alternatives to the commercial anti-bacterial agents [18,54,55]. In this work, an anti-bacterial film was prepared by incorporating the Wells-Dawson polyoxometalate into the κ-carrageenan matrix using the tape-casting method. The prepared Car/POM film demonstrated good bactericidal properties against common foodborne bacteria including *E. coli* and *S. aureus*, as demonstrated in both Kirby-Bauer disc diffusion assay and carrier surface disinfection tests. In addition, our obtained results also suggested that addition of polyoxometalates at low concentration (≤4 mg/mL) only slightly changed the microstructure of films, while Carr/POM (at 8 mg/mL) film showed desirable anti-bacterial activity with acceptable decreases in the water content and tensile strength. Admittedly, further studies are needed before the polyoxometalate-based film could eventually be used as commercial active food packaging in the food industry. For instance, besides the mandatory safety assessment, it is also necessary to assess the impact of this film on the appearance and texture of foods. In conclusion, our current work suggested that polyoxometalates had great application potential as an active agent to improve the anti-bacterial effect of the carrageenan film.

## Figures and Tables

**Table 1:** Effect of the concentration of POM on the opacity and color of Carr/POM anti-bacterial films.

Concentration (mg/mL)	Opacity	L	a	b	∆E
0	22.704 ± 5.4115 ^a^	20.960 ± 2.588 ^a^	114.353 ± 5.542 ^a^	−41.59 ± 2.326 ^a^	123.509 ± 5.455 ^a^
1	23.028 ± 0.260 ^b^	20.857 ± 2.051 ^a^	115.267 ± 5.832 ^a^	−41.817 ± 2.353 ^a^	124.405 ± 5.827 ^a^
2	36.968 ± 2.980 ^b^	23.423 ± 2.319 ^a^	119.523 ± 9.984 ^a^	−43.720 ± 4.720 ^a^	129.412 ± 11.189 ^a^
4	90.031 ± 4.783 ^c^	34.433 ± 4.702 ^b^	90.787 ± 7.040 ^b^	−33.263 ± 3.178 ^b^	102.779 ± 6.176 ^b^
8	82.047 ± 2.024 ^c^	40.653 ± 1.620 ^c^	54.393 ± 1.747 ^c^	−18.927 ± 1.234 ^c^	70.526 ± 0.816 ^c^

Different lowercase letters represent significant differences, and the same lowercase letters represent no significant differences (*p* < 0.05).

## Data Availability

Data is contained within the article.
